# What is the mechanism for persistent coexistence of drug-susceptible and drug-resistant strains of *Streptococcus pneumoniae*?

**DOI:** 10.1098/rsif.2009.0400

**Published:** 2009-11-25

**Authors:** Caroline Colijn, Ted Cohen, Christophe Fraser, William Hanage, Edward Goldstein, Noga Givon-Lavi, Ron Dagan, Marc Lipsitch

**Affiliations:** 1 Department of Engineering Mathematics, University of Bristol, Bristol, UK; 2 Center for Communicable Disease Dynamics, Department of Epidemiology, Harvard School of Public Health, Boston, MA, USA; 3 Department of Immunology and Infectious Diseases, Harvard School of Public Health, Boston, MA, USA; 4 Division of Global Health Equity, Brigham and Women's Hospital, Boston, MA, USA; 5 Department of Infectious Disease Epidemiology, Imperial College London, UK; 6 Medical Research Council Centre for Outbreak Analysis and Modelling, Imperial College London, UK; 7 Pediatric Infectious Diseases Unit, Soroka University Medical Center, Beer Sheva, Israel

**Keywords:** epidemiology, drug resistance, mathematical model, coexistence

## Abstract

The rise of antimicrobial resistance in many pathogens presents a major challenge to the treatment and control of infectious diseases. Furthermore, the observation that drug-resistant strains have risen to substantial prevalence but have not replaced drug-susceptible strains despite continuing (and even growing) selective pressure by antimicrobial use presents an important problem for those who study the dynamics of infectious diseases. While simple competition models predict the exclusion of one strain in favour of whichever is ‘fitter’, or has a higher reproduction number, we argue that in the case of *Streptococcus pneumoniae* there has been persistent coexistence of drug-sensitive and drug-resistant strains, with neither approaching 100 per cent prevalence. We have previously proposed that models seeking to understand the origins of coexistence should not incorporate implicit mechanisms that build in stable coexistence ‘for free’. Here, we construct a series of such ‘structurally neutral’ models that incorporate various features of bacterial spread and host heterogeneity that have been proposed as mechanisms that may promote coexistence. We ask to what extent coexistence is a typical outcome in each. We find that while coexistence is possible in each of the models we consider, it is relatively rare, with two exceptions: (i) allowing simultaneous dual transmission of sensitive and resistant strains lets coexistence become a typical outcome, as does (ii) modelling each strain as competing more strongly with itself than with the other strain, i.e. self-immunity greater than cross-immunity. We conclude that while treatment and contact heterogeneity can promote coexistence to some extent, the in-host interactions between strains, particularly the interplay between coinfection, multiple infection and immunity, play a crucial role in the long-term population dynamics of pathogens with drug resistance.

## 1. Introduction

The use of antimicrobial drugs has led to the emergence and spread of resistant strains of nearly every bacterial pathogen. For some antimicrobial agents and bacterial species, resistant strains now comprise nearly 100 per cent of the bacterial population. A notorious example is *Staphylococcus aureus*, which became almost universally resistant to penicillin within a few years of the introduction of this drug. Development of extended spectrum drugs such as oxacillin and methicillin addressed the emergence of these penicillin-resistant strains of *S. aureus*, but resistance to these antibiotics is now common, and their effectiveness is declining (McDonald 2006). For other bacterial species, resistant strains have become clinically important, but seem to remain at intermediate frequencies, neither dwindling to zero nor reaching 100 per cent. Examples include vancomycin resistance in *Enterococcus* (McDonald 2006), sulphonamide resistance in *Escherichia coli* (Enne *et al*. 2001) and, seemingly, penicillin (and other drug) resistance in *Streptococcus pneumoniae*.

From an evolutionary perspective, it is easy to explain why a gene would have a frequency near zero—if it is on average deleterious; or near 100 per cent—if it is on average beneficial. In the simplest models of competition between drug-susceptible and drug-resistant strains, *competitive exclusion* is predicted, with the ‘fitter’ strain—that with the higher basic reproductive number *R*_0_ at a given level of antimicrobial use—winning (Lipsitch 2001). A prevalence near 0 or 100 per cent may be attributable to mutation–selection balance. This would occur if (for example) the resistant strain has lower fitness in the population as a whole, but reappears at some frequency owing to mutation or resistance gene acquisition (Cohen & Murray 2004). Alternatively, the resistant strain may be selectively favoured but occasionally lose its resistance determinant (Vilhelmsson *et al*. 2000), producing a frequency near 100 per cent. For hospital-acquired pathogens, a migration–selection balance could maintain polymorphism, if a resistant strain is selectively favoured in the hospital where antibiotic usage is high but constantly ‘diluted’ by the influx of sensitive strains, that are favoured in the community, as new patients are admitted (Lipsitch *et al*. 2000). For commensal organisms like *E. coli* that form a permanent part of the normal flora, it is possible that selection by antimicrobial use within hosts may be countered by novel introduction of (mainly drug-sensitive) strains from food or other sources—a within-host form of migration–selection balance (Levin *et al*. 1997; Stewart *et al.* 1998).

For *S. pneumoniae*, a common but not universal inhabitant of the nasopharyngeal flora, none of these explanations seems to fit in a simple way. In §2, we make the case that *S. pneumoniae* is showing long-term (in this case over many years, up to a decade) persistence of both drug-sensitive and drug-resistant strains. The explanation of mutation–selection balance (or recombination–selection balance, since pneumococci undergo very high rates of recombination; Feil *et al*. 2001) is implausible because typical frequencies of resistance in populations are not near zero or one, but rather in the tens of per cent, and because these frequencies can vary so rapidly (Dagan *et al*. 2008). The explanations relevant to hospital-acquired pathogens or to permanent commensals are not applicable because the duration of pneumococcal colonization is of the order of weeks to a few months (Bogaert *et al*. 2004; Hogberg *et al*. 2007); the prevalence is well below 100 per cent in developed countries (Bogaert *et al*. 2004); and there is no known source of ‘influx’ of drug-sensitive strains, since it is an obligate human colonizer (Bogaert *et al*. 2004). Nonetheless, as will become clear below, we consider variations on these themes as possible explanations of the coexistence of resistant and susceptible pneumococci.

In a recent paper (Lipsitch *et al*. 2009), we proposed that when using mathematical models to understand the mechanisms of coexistence of pathogen strains in a host population, the underlying model should not create ‘coexistence for free’. More technically, when applied to two biologically indistinguishable strains, the model's ecological dynamics should be independent of the strain composition of the population, and the model should not predict a stable equilibrium frequency of the two strains, but rather permit any long-run frequency given some possible initial conditions. In that paper, we define sufficient conditions under which a model meets these criteria when applied to indistinguishable strains; we call such models ‘structurally neutral models’. Here, our goal is to start from simple structurally neutral models, and ask whether they consistently predict stable coexistence when applied to resistant and sensitive strains, which are of course not indistinguishable. We initially consider five models (A–E), each of them structurally neutral. Apart from the first, each one incorporates some mechanism that may potentially promote coexistence of competing strains, and we explore the ability of each model to do so. Finally, we consider the possibility that the two strains experience some degree of strain-specific immunity, a feature that breaks the structural neutrality of the model, but which may possibly reflect biological reality. Under these circumstances, we repeat analyses of models A–E and find that the conditions for coexistence are considerably expanded. A number of factors, such as trade-offs in transmissibility and virulence, spatial heterogeneity or different selective pressures in weakly interconnected subpopulations, contact structure, heterogeneous host susceptibility and mechanisms for sustained non-equilibrium coexistence are all likely to play a role in maintaining strain diversity. We developed models A–E in light of several of these.

Before describing the models, we review the evidence supporting long-term, apparently stable coexistence of drug-sensitive and drug-resistant strains of *S. pneumoniae*.

## 2. Evidence for stable coexistence of drug-resistant and drug-sensitive *streptococcus pneumoniae*

Resistant strains of *S. pneumoniae* have been reported to date for every important drug class used to treat it, with the notable exception of vancomycin. In some settings, there is evidence that the prevalence of resistance has been increasing in recent years. Among invasive isolates in the USA prior to the introduction of the pneumococcal conjugate vaccine (which reduced resistance by targeting mainly those serotypes most associated with resistance; Kyaw *et al*. 2006), there was a trend of increasing prevalence of resistance or non-susceptibility to penicillin, cephalosporins, macrolides and trimethoprim sulfamethoxazole (Whitney *et al*. 2000; McCormick *et al*. 2003). In Canada, similar trends were observed for penicillin non-susceptibility and macrolide resistance, though not for trimethoprim sulfamethoxazole (Powis *et al*. 2004). The absolute increase in the prevalence of resistance was of the order of 1–4% per year for each of these populations. In The Netherlands between 1994 and 1999, most pneumococci remained susceptible to both penicillin and macrolides, but there was a modest upward trend of less than 1 per cent per year in absolute prevalence (de Neeling *et al*. 2001).

In other populations, there is evidence that the prevalence of resistance has been at a plateau for a number of years. The European Antimicrobial Resistance Surveillance System (EARSS) has reported antimicrobial susceptibilities for pneumococci since 2001. In the EARSS report up to 2006, there were 21 countries that submitted an average of 100 or more isolates per year for susceptibility testing, for some period starting as early as 2001 and ending in 2006 (Anonymous 2007). Among these 21 countries, there were only six showing evidence of a trend in the prevalence of penicillin non-susceptibility (decreasing in four, increasing in two), four with a significant trend in the prevalence of penicillin resistance (increasing in two and decreasing in two) and eight with a trend in macrolide resistance (increasing in six and decreasing in two). Perhaps the longest time series on resistance we have found in the literature is a large but non-systematic sample of clinical pneumococci from Spain, whose susceptibility patterns have been traced since as early as 1979. Non-susceptibility to penicillin remained roughly fixed between the late 1980s and 1996, fluctuating around 60 per cent in non-invasive isolates and around 40 per cent in invasive ones (Fenoll *et al*. 1998); a subsequent report showed this trend continued (with perhaps a decline of less than 5%) through 2001 (Fenoll *et al*. 2002). The prevalence of cefotaxime resistance remained around 20 per cent from the late 1980s through 2001 (Fenoll *et al*. 1998, 2002), though erythromycin resistance was increasing during the first reporting period, up through 1996 (Fenoll *et al*. 1998); a 2004 sample from the same group (Calatayud *et al*. 2007), combined with EARSS data from Spain (Anonymous 2007), suggests that erythromycin resistance plateaued by the early part of this decade and may be starting to decline modestly.

Collectively, these studies show that for the most commonly used antimicrobial classes—penicillins, cephalosporins and macrolides—resistance is changing little, if at all, over the decadal scale in many populations, though in the USA, Canada and Spain in the 1990s, there was a distinctive upward trend in macrolide resistance. One caveat in interpreting these studies is that essentially all of them use isolates from invasive disease, rather than nasopharyngeal carriage, which is the ‘reservoir’ for infection and the site of most selection by antimicrobials (Bogaert *et al*. 2004; Dagan & Lipsitch 2004). However, invasive isolates are a subset of carriage strains, hence coexistence of resistant and sensitive strains among invasive isolates suggests the same among carriage isolates, and broad patterns of resistance appear similar among carriage and invasive isolates (Huang *et al*. 2005; Kyaw *et al*. 2006).

How can we reconcile this common observation with the model-based prediction that, under a constant level of antimicrobial use, the rate of change in the prevalence of resistance should be either constantly positive (leading eventually to 100% prevalence) or constantly negative (leading eventually to 0% prevalence)? There are several possibilities. One is that as resistance has grown, there has been a corresponding decline in antimicrobial use, so that what we see is essentially the peak of resistance, which will soon decline as antimicrobial use reaches sustained lower levels. This interpretation is hard to reconcile with several facts. First, within Europe, there is enormous (approximately threefold) variation between countries in the intensity of antimicrobial use, which correlates closely with the prevalence of penicillin resistance in pneumococci (Goossens *et al*. 2005); second, the apparent plateaus in resistance appear to be scattered throughout the low-, middle- and high-use countries, while the countries in which resistance is increasing tend often to be those with low levels of antimicrobial use and low levels of resistance (Anonymous 2007). Thus it is difficult to believe that the plateaus mainly correspond to those areas that are changing their level of use from a high level that promotes resistance to a low level that opposes its rise.

A second possible reconciliation is that the natural time scale for trends in resistance is very long, and that apparent plateaus are concealing very slow increases. This seems unlikely for two reasons. First, one can make a crude calculation that with approximately 0.5–1.5% of the population of a European country taking penicillins each day (Goossens *et al*. 2005), the incidence of new prescriptions might be of the order of 3–6% per month, and probably higher in the age groups that carry pneumococci. Since the duration of pneumococcal carriage is of the order of 1–2 months (Hogberg *et al*. 2007), use of penicillins arguably reduces the expected duration of pneumococcal carriage for sensitive strains by roughly 3–12%. A resistant strain, which avoided that clearance, might therefore have a roughly 3–12% advantage in transmission, excluding any fitness cost associated with penicillin resistance (Trzcinski *et al*. 2006). Given that the ‘generation time’ of pneumococcal carriage is about a month or two, a fitness advantage of even 1 per cent for resistant strains should result in an about 1 per cent per month exponential increase in the prevalence odds of resistant strains (Lipsitch 2001). Here (unlike the figures above) we are describing proportional increases, not absolute increases, so a 1 per cent per month exponential increase corresponds to a doubling in about 5–6 years. Thus, under fairly conservative assumptions, the time scale of change should be faster than what we currently observe. Moreover, recently published data from Israel indicate that the prevalence of resistance can change even more rapidly, with resistance fluctuating roughly two- to threefold in a seasonal cycle, increasing to a winter peak (as antibiotic use increases) and declining to a summer trough (Dagan *et al*. 2008). Thus, it is difficult to believe that the slow changes observed over years to decades reflect an inability of pneumococcal populations to respond rapidly to selection for resistance.

If plateaus in the prevalence of resistance are real for *S. pneumoniae*, and they cannot be explained either by declines in antimicrobial use or by a naturally slow time scale for changes in the prevalence of resistance, we believe that existing models are missing something critical in the transmission dynamics of resistant organisms. Under this hypothesis, a model is needed in which approximately constant levels of antimicrobial use lead to stable equilibria with a prevalence of resistance that is neither almost zero nor almost 100 per cent, i.e. in which competitive exclusion is not the expected outcome of competition between resistant and sensitive strains. In the sections that follow, we consider this possibility in greater detail and explore several models for their ability to replicate the apparently stable, but intermediate, prevalence of resistance observed in many pneumococcal populations.

## 3. Methods

### 3.1. Five model structures

We develop five models of *S. pneumoniae* colonization. Because colonization is much more common than invasive disease, and colonized persons are thought to be the main source of pneumococcal transmission, we ignore disease in these models and consider colonization only; we thus use the term ‘infection’ interchangeably with colonization.

In each model there are two strains, one that is sensitive to a particular antibiotic and one that is resistant. In reality, a resistant strain may retain some susceptibility to that drug. In our models, each strain has a clearance rate independent of treatment, so that if we were to model treatment effective against both strains, this could be modelled as an increase in the overall clearance rate. We therefore model the differential effect of therapy on the two strains as treatment that has no effect on the strain designated as ‘resistant’.

In each of our models, infection by both strains is possible. The extent to which dual infection occurs in the models depends on whether the presence of a resident strain inhibits acquisition of a second strain upon subsequent exposure, and (in one case) on the relative fitness of the strains. We do not explicitly model the dynamics of host immunity (which may in principle take the form of reduced acquisition rates and/or reduced duration of carriage); rather, the assumed values of transmission and clearance rates are meant to average over more and less immune members of the population.

The first model considered is a simple model in which individuals may be susceptible, infected (and infectious) with either strain or dually infectious with both strains. The second is an extension of this simple model, with explicit accounting for those individuals currently on antibiotic treatment. Similar ‘habitat heterogeneity’ has been shown in ecological models to promote coexistence of competing populations (Tilman & Pacala 1993). The third model is also an extension of the first, and includes a ‘day-care’ environment that is attended by a portion of the population and that is characterized by both high transmission and high rates of treatment. The day care thus provides a different form of habitat heterogeneity, and can also be thought of as a school or workplace or any environment in which there is more contact and a greater rate of treatment than in the general population. The fourth model allows the possibility that if there is competition between low fitness strains and a higher fitness strain, the higher fitness strain may superinfect hosts colonized with the low fitness strain and may subsequently outcompete the low fitness strain within these dually infected hosts. The fifth model borrows some inspiration from prior models of long-term commensals like *E. coli* (Levin *et al*. 1997; Stewart *et al*. 1998), and permits the persistence of subpopulations of drug-sensitive strains within individuals carrying mainly the resistant strain, and vice versa. Unlike those prior models, it is a pure transmission model that does not assume entry of novel strains from outside the model, and it allows clearance of *S. pneumoniae* from a host. In each model, as in the framework given in Lipsitch *et al*. (2009), it is possible to be colonized with either the drug-sensitive or the drug-resistant strain alone, with both or with neither.

**Figure 1.**
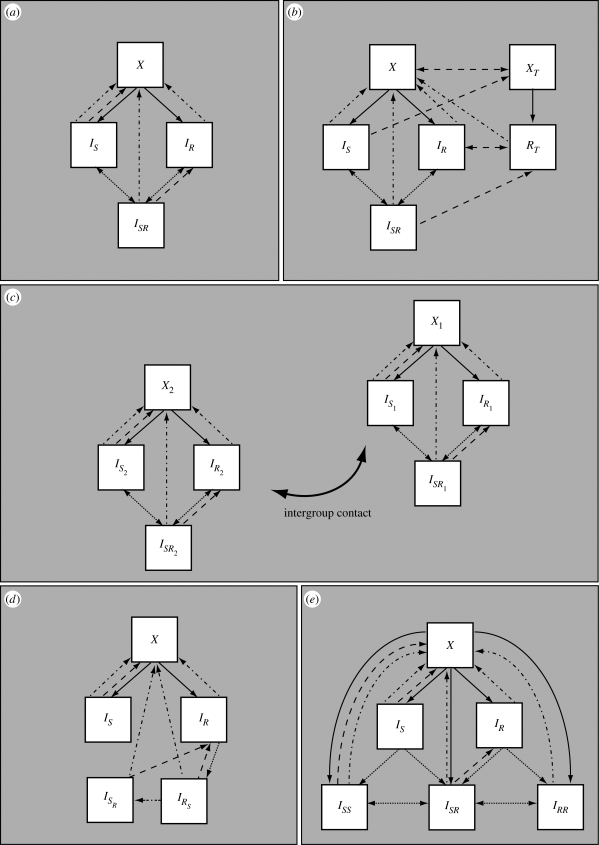
Schematic of the models. *X* indicates susceptible, *I*_*S*_ infectious with the sensitive strain and *I*_*R*_ with the resistant strain, while *I*_*SR*_ indicates dually infected individuals. (*a*) In model A, dotted arrows indicate reinfection, dot-dashed arrows indicate natural clearance of infection and dashed arrows indicate treatment (and its waning, in model B). (*b*) In model B, the subscript *T* indicates the treated class. (*c*) In model C, the subscript 1 indicates the general population while 2 indicates the day-care/school subpopulation. (*d*) In model D, *I*_*SR*_ specifies dually infected individuals who have predominant sensitive infections and *I*_*RS*_ specifies dually infected individuals who have predominantly resistant infections. (*e*) In model E, in addition to the singly infected classes, there are three dually infected classes *I*_*SS*_, *I*_*SR*_ and *I*_*RR*_.

Model A is a simple model with single and dual colonization, illustrated in figure 1*a*. This basic model serves as a basis for constructing the more complex models B–E described below. Model A contains four classes of individuals as described above: *X* (susceptible), *I*_*S*_ (infected with the sensitive strain), *I*_*R*_ (infected with the resistant strain) and *I*_*SR*_ (infected with both strains). Infection occurs at a rate proportional to a transmission rate constant *β*_*s*_ or *β*_*r*_ for the sensitive and resistant strains, respectively, and to the prevalence of singly or dually infected hosts. Dually infected individuals may transmit either the susceptible or resistant strain upon contact, with equal probability, so that the forces of infection are *β*_*s*_(*I*_*S*_ + *qI*_*SR*_) for the sensitive strain and *β*_*r*_(*I*_*R*_ + *qI*_*SR*_) for the resistant strain; throughout this paper, we fix *q* = 1/2 to indicate that dually infected hosts are equally infectious as the average infectiousness of singly infected hosts with the sensitive or resistant strain alone. An individual in class *I*_*S*_ can move to class *I*_*SR*_ upon reinfection with the resistant strain, but reinfection is somewhat less efficient than primary infection, occurring at a *per capita* rate 1/2 ≤ *k* ≤ 1 times as great. We chose to have 1/2 as the lower bound for *k* to model the assumption that infection only confers partial immunity and to allow reinfection and coinfection to play a role in the model dynamics. Likewise, individuals in class *I*_*R*_ are partially protected against reinfection with the sensitive strain. In these models, reinfection results in partial strain replacement within the host; singly infected individuals move to the dually infected class while dually infected individuals may move back to either class *I*_*S*_ or *I*_*R*_ upon reinfection, indicating replacement of one strain by a ‘new copy’ of the other strain. With probability *c* = 1/2, a reinfection of a dually infected individual replaces the same strain, resulting in no change. The choice *c* = *q* = 1/2 guarantees the structural neutrality of the base model. Natural clearance occurs at rate *u* from all infected states and results in the return of individuals to the class of uninfected individuals. We model treatment at rate τ as taking individuals infected with the sensitive strain back to the uninfected class, and dually infected individuals to the resistant class, as treatment cures the sensitive part of the infection. We do not model acquired resistance (the de novo appearance of a resistant variant upon treatment of a sensitive strain) because we are primarily concerned with resistance to penicillins and macrolides, each of which requires the import of a novel gene or allele from an already-resistant strain by transformation (or transconjugation). This event occurs very rarely, since it requires co-colonization with a sensitive recipient and a resistant donor, combined with selection for resistance that preferentially selects a transformant or a transconjugant (rather than just the donor).

The model is given by equation (3.1). The fact that the model is neutral for identical strains means that we are not introducing coexistence ‘for free’ by building it into the model structure in a fundamental way.
3.1
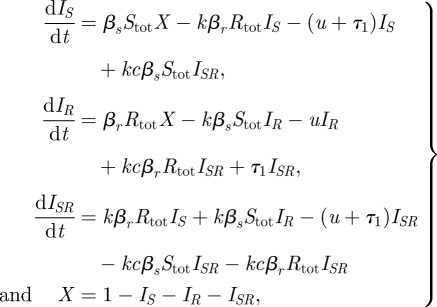

where *S*_tot_ = *I*_*S*_ + *qI*_*SR*_ and *R*_tot_ = *I*_*R*_ + *qI*_*SR*_.

Model B (figure 1*b*) expands upon model A by including an explicit class of treated persons, whereas treatment in model A was an instantaneous event. Treatment may be given to susceptibles, whereupon they enter the treated susceptible class (*X*_*T*_) and cannot be successfully infected by the drug-sensitive strain. Individuals infected with the sensitive strain and given treatment move to the *X*_*T*_ class. Individuals with the resistant strain who are being treated (class *R*_*T*_) cannot be productively infected by the sensitive strain. Dually infected individuals given treatment move to the *R*_*T*_ class. Individuals end treatment and exit classes *X*_*T*_ and *R*_*T*_ at rate *w*, whereupon individuals move back to *X* and *I*_*R*_, respectively. The dynamics of reinfection are the same as in model A. Model B is given by equation (3.2) and reduces to model A in the limit where the two strains are biologically identical, because it does not make sense to treat only one of the two identical strains, so either the treatment rate must be 0 (reducing the model to model A) or treatment must apply equally well to both strains, so that the model would need a treated compartment for both.
3.2
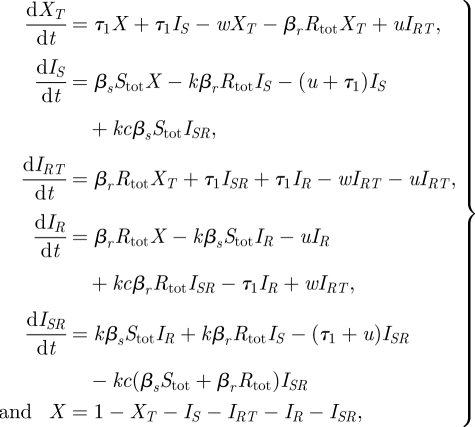

where *S*_tot_ = *I*_*S*_ + *qI*_*SR*_ and *R*_tot_ = *I*_*R*_ + *I*_*RT*_ + *qI*_*SR*_.

Model C (figure 1*c*) expands on model A in a different direction, by including two subpopulations with different transmission dynamics. In model C, the population is heterogeneous, with a proportion *h* experiencing both a higher rate of transmission and a higher frequency of antimicrobial treatment than the remainder of the population; the two groups might represent younger versus older children or day-care attendees versus non-day-care attendees. The model structure incorporates two copies of model A: one for the general population (group 1), and one for the day-care environment (group 2), in which both the contact rate and the treatment rate are higher. The two groups are coupled via the force of infection terms, which summarizes the extent to which contact between the groups is assortative or random, the portions of the population in each group and the different contact rates in the groups. As in model A, the limiting case of indistinguishable strains for this model would mean that transmission rates for the strains are the same and the treatment rate is 0, and this would result in a structurally neutral model. The model is given by
3.3
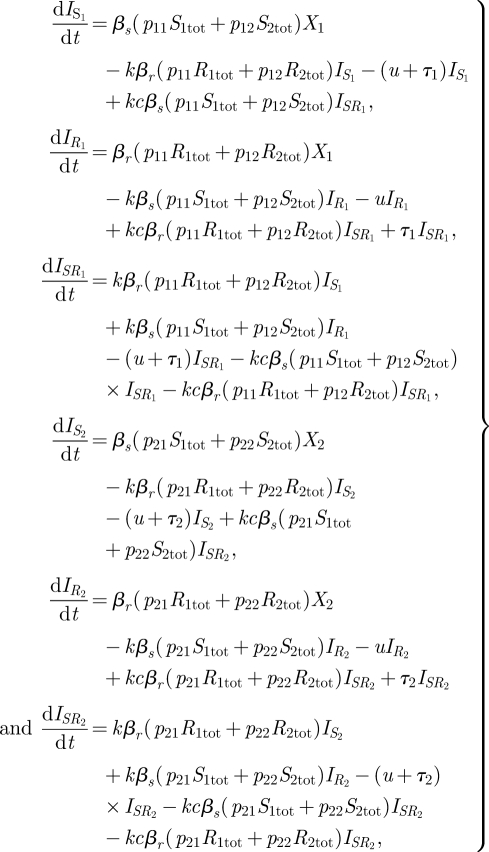

where *S*_1tot_ = *I*_*S*__1_ + *qI*_*SR*__1_ and *R*_1tot_ = *I*_*R*__1_ +*qI*_*SR*__1_ and similarly for *S*_2tot_ and *R*_2tot_.

Here, *p*_*ij*_ (*i*, *j* ∈ {1,2}) represents the rate at which individuals of group *i* receive potentially infectious contacts from group *j*, given by the following equation:
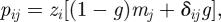

where *z*_*i*_ represents the *per capita* rate at which individuals of group *i* make infectious contact and δ_*ij*_ is 1 if *i* = *j* and 0 otherwise. The number *g* ∈[0,1] measures the degree of assortative mixing, with *g* = 0 corresponding to random mixing and *g* = 1 corresponding to fully assortative mixing. In these calculations, *m*_1_ = (1 − *h*)*z*_1_/((1 − *h*)*z*_1_ + *hz*_2_) is the proportion of all contacts made by members of group 1 (the low-contact group) and *m*_2_ = 1 − *m*_1_ is the proportion of contacts made by members of group 2 (the high-contact group).

Model D (figure 1*d*, equations (3.4)) departs from model A in two ways. First, the dually infected category is now split into two, those who carry primarily the sensitive strain with a small resistant subpopulation (*I*_*SR*_), and those who carry primarily the resistant strain with a small sensitive subpopulation (*I*_*RS*_). Each of these types of hosts transmits only the predominant strain. Second, this model assumes that reinfection can occur only when a strain of higher ‘fitness’ within the host encounters a host carrying a strain of lower fitness. Here fitness is defined as competitive ability within the host in the absence of antibiotics, and consistent with prior experimental data, we assume that the resistant strain is of lower fitness than the sensitive strain (Andersson & Levin 1999; Trzcinski *et al*. 2006). Accordingly, there may be reinfection of individuals carrying the less fit resistant strain, but there is no reinfection of those carrying the drug-sensitive strain. Additionally, this model stipulates that within coinfected individuals, the more transmissible strain can outcompete the less transmissible strain (at rate *ξ*) so that the more transmissible becomes the dominant one. We also assume that when evaluating strain coexistence at the population level, a dually colonized individual is measured as having only the predominant strain. This assumption is consistent either with what one would expect from invasive isolates (which are usually clonal, representing invasion of a particular strain from the nasopharynx) or from carriage isolates as typically characterized by growing and characterizing a single colony-forming unit. This model reduces to a structurally neutral model when the strains are identical because in this case, neither strain has higher fitness, so there can be no reinfection, and the model collapses into model A with *k* = 0.
3.4
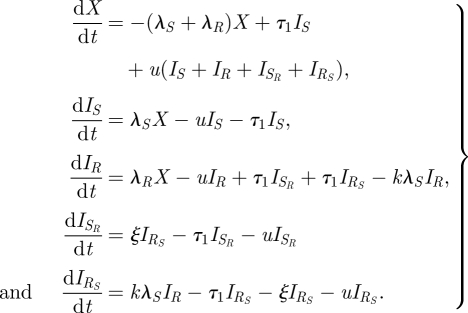

The forces of infection are written as *λ*'s and are given by
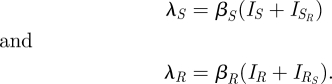



Finally, model E (figure 1*e*, equations (3.5)) expands model A in two different ways. Here, reinfection may result in individuals having more than one ‘copy’ of a single type of infection, in analogy to the state of simultaneous infection with *S* and *R*. This model has six states in total, and three dually infected states. Furthermore, in this model, simultaneous transmission of a dual infection may occur when a susceptible individual is in contact with a dually infected individual; this is a departure from the assumptions in previous models, where only one strain could be transmitted at a time. In this case, a single infection (resulting in the individual moving from *X* to *I*_*S*_ or *I*_*R*_) occurs with probability *ρ*_single_. When a dually infected *I*_*SR*_ individual is in contact with a susceptible individual and a single infection is to be transmitted, *S* is transmitted with probability *ρ*_*s*_ and *R* is transmitted otherwise. In this model, we do not explicitly model changes of state within a colonized host, except through reinfection or clearance; we do not allow for the possibility that (say) an *R* host can become an *RR* host by growth of the *R* strain. This model is also a structurally neutral model for biologically identical strains.
3.5
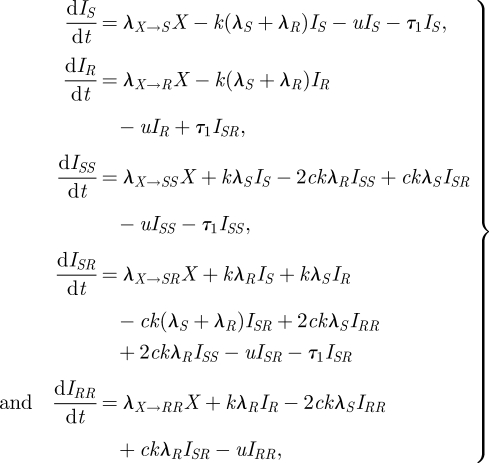

where *X* = 1 − *I*_*S*_ − *I*_*R*_ − *I*_*SS*_ − *I*_*SR*_ − *I*_*RR*_, and the forces of infection are defined as follows:
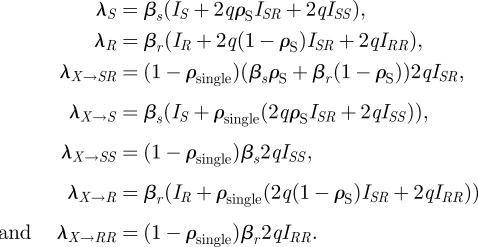

These expressions allow for different contributions to the forces of infection from singly and dually infected individuals. Those who are dually infected are *q* times as infectious with each of their strains as those who are singly infected; in these simulations, we use *q* = 1/2 under the assumption of equal infectiousness for dually and singly infecteds, though this choice is not essential to retain the neutrality of the model (Lipsitch *et al*. 2009).

### 3.2. Parameter values

To assess the extent to which coexistence is a ‘generic’ outcome of these models, we defined a set of plausible ranges for the values of each of their parameters. For parameters that were common to more than one model, we set the plausible ranges the same in each model. Where data were available to constrain the plausible range of parameters, we used the data to do so; however, the values of many of these parameters are difficult or impossible to measure, in which case we set a broad range of ‘plausible’ values. For each parameter set, we ran the model to near equilibrium (which was achieved by the end of 1000 simulated months) and asked whether the model exhibited elimination of one or the other strain or whether there was stable coexistence of strains. Stable coexistence was defined as the final frequency of the sensitive strain (among all colonized hosts) between 1 and 99 per cent; frequencies outside this range were defined as showing competitive exclusion. To assess the frequency of stable coexistence in parameter space, we created 10 000 parameter sets, each by an independent uniform draw of all parameters from their plausible ranges given in table 1; this choice was made for simplicity and because in general we do not have data to inform a different distributional estimate. The ranges for our parameters affect the portion of simulations with coexistence; as a simple example, the ranges are chosen so that *R*_0_ is greater than one for both strains; if we had allowed parameter ranges such that the reproductive number was less than one, we would have seen some portion of simulations with no infection at all. Since the parameter ranges are fixed across the model structures, while the frequency of coexistence in the simulations may not reflect that which would be statistically observed owing to the real parameters being distributed in a non-uniform fashion or over ranges other than those chosen here, it is the qualitative level of coexistence and particularly the comparison between model structures that is our focus.

**Table 1.** Parameters in the models. Where a range is given, values are uniformly distributed across that range. Where a single value is given, the parameter was kept at that value.

parameter	range	description	rationale
*β*_*r*_/*β*_*s*_	0.75–1	ratio of transmission parameters for resistant : sensitive strains	assumes resistant strain is never more transmissible than the sensitive strain, but that the minimum possible *R*_0__*R*_ for resistant strain is 1
*β*_*s*_	1.33–4.33	transmission parameter for the sensitive strain	*R*_01_ range from 1.33 to 4.33 and *R*_02_ range from 1 to 3.25. Based on assuming an SIS model and observed prevalence of carriage in the range of 30–80% (Bogaert *et al*. 2004)
*τ*_1_/*τ*_2_	1–6	relative treatment rate in the high-treatment environment (model C only)	assumption: estimates and 95% confidence intervals for estimates comparing infants in day care to other infants in Scandinavia are 2.43 (1.34–4.41) (Hedin *et al*. 2007) and 2.0 (1.7–2.3) (Thrane *et al*. 2001). We consider a broader range because slightly older children may use less antibiotics while still contributing to transmission
*τ*_1_	1/12 to 1/3 per month	treatment rate in the lower treatment compartments	Finkelstein *et al*. (2001), using the rates in under-3 and under-6 children, respectively
*k*	1/2 to 1	1-partial immunity	assumption
*u*	1 per month	clearance rate	Ekdahl *et al*. (1997), Hogberg *et al*. (2007)
*c*	1/2	fraction of duals returning to *I*_*S*_ (*I*_*R*_) upon reinfection with *S* (*R*)	necessary for structurally neutral model in simple model when strains are indistinguishable (Lipsitch *et al*. 2009)
*q*	1/2	relative infectiousness with each strain for dually infecteds (*I*_*SR*_); enforces that dually infecteds as infectious as singly infecteds	necessary for structurally neutral model in simple model when strains are indistinguishable (Lipsitch *et al*. 2009)
*z*_2_/*z*_1_	1–5	relative contact rate in the high-contact environment (model C only)	assumption
*z*_1_	1	base contact factor in the day care/school/work model (model C)	null value
*h*	0.05–0.5	portion of population in the high-contact compartment (model C)	widest reasonable range
*g*	0–1	type of mixing in model C. 0 is random mixing, 1 is assortative mixing	full range
*w*	3–8 per month	rate of treatment waning in treated class model (model B)	length of treatment: approximately 4–10 days
*ρ*_single_	0.1, 0.5, 0.9	probability that a single (rather than dual) infection is transmitted (model E)	symmetric value 0.5. Explored 0.1 and 0.9 effect on coexistence
*ρ*_*s*_	0.5–1	probability that *S* is transmitted when a dually infected individual transmits single infection (model E)	symmetric value 0.5. Larger values model a fitness cost of resistance through giving an advantage to strain *S*
*ξ*	1/10 to 10 per month	within-host takeover rate at which *I*_*RS*_ → *I*_*SR*_ (model E)	range of two orders of magnitude with concentration at low takeover rate (this parameter is sampled uniformly on log scale)

## 4. Results

### 4.1. Findings from models A–E

Stable coexistence is possible in each model, but is not typical in the baseline case of model A; in appendix A, we show a bifurcation diagram for this model, indicating that there is a thin band in parameter space in which stable coexistence is possible (figure 4).

**Figure 2.**
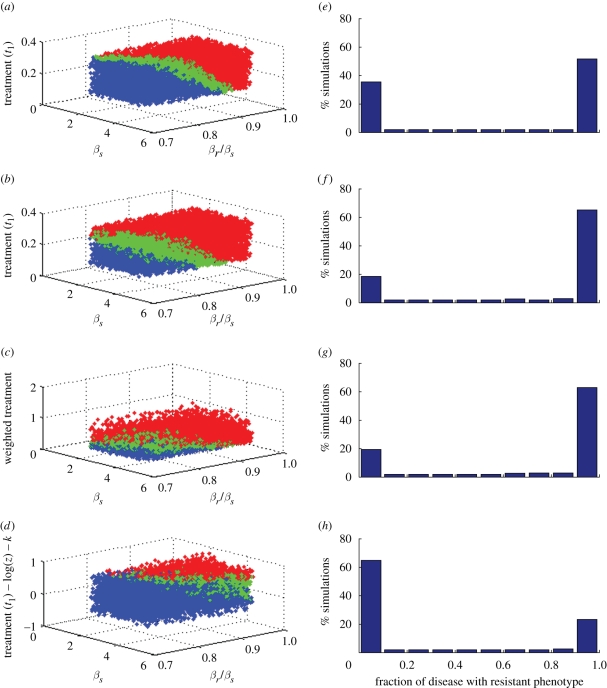
Scatter plots of the simulations in the (*a*) simple (model A), (*b*) treated (model B), (*c*) day care/school/work (model C) and (*d*) within-host strain takeover models (model D) showing their outcomes at equilibrium and (*e*–*h*) histograms of the fraction of infection that is resistant at equilibrium. Green, coexistence; blue, all disease is strain *S*; red, all disease is strain *R*.

Figure 2*a*–*d* shows the simulations in models A–D according to their eventual outcome: coexistence (green), elimination of the drug-sensitive strain (red) or elimination of the drug-resistant strain (blue). The three parameters on the axes are *β*_*s*_ (the transmission parameter of the sensitive strain), the ratio of transmission parameters of the resistant to sensitive strain *β*_*r*_/*β*_*s*_ and the rate of treatment (or, in the case of model C, a weighted treatment term [(1 − *h*)*τ*_1_ + *hτ*_2_] and in the case of model D, a modified treatment term that also reflects the importance of the rate of within-host takeover and partial immunity to reinfection: *τ*_1_/*u* − log(*ξ*/*u*) − *k*). Though there are more parameters that vary between simulations, these variables effectively separate the simulations by their qualitative outcomes in each of these models. We observe that when the relative transmissibility of the resistant strain is higher, the sensitive strain is eliminated. When the level of treatment is higher, the resistant strain takes over at lower levels of relative transmissibility because increased treatment provides a greater competitive advantage for resistance. When the reproductive numbers of the two strains (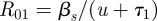 and 
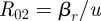
 in the simple and treated class models) are approximately equal, coexistence occurs. This accounts for the placement of the green near-planar areas in the figure; see also figure 4 in appendix A.

Over the sampled parameter ranges, approximately 16 per cent of the simulations exhibit coexistence in the simplest model, model A, with slightly higher fractions of simulations exhibiting coexistence in model B (19%; the treated class model) and model C (20%; the day-care model). Figure 2*e*–*h* shows a histogram for each model depicting the fraction of carried strains that were resistant at equilibrium over 10 000 simulations.

Model D, in which the sensitive strain is more transmissible and can reinfect a host carrying the resistant strain (but not vice versa), reveals the lowest levels of coexistence over the ranges of parameters sampled (approx. 10%). Figure 2*d*,*h* shows that the sensitive strain is highly favoured, and the probability that the resistant strain will eliminate the sensitive strain is lower than in the other models we consider.

**Figure 3.**
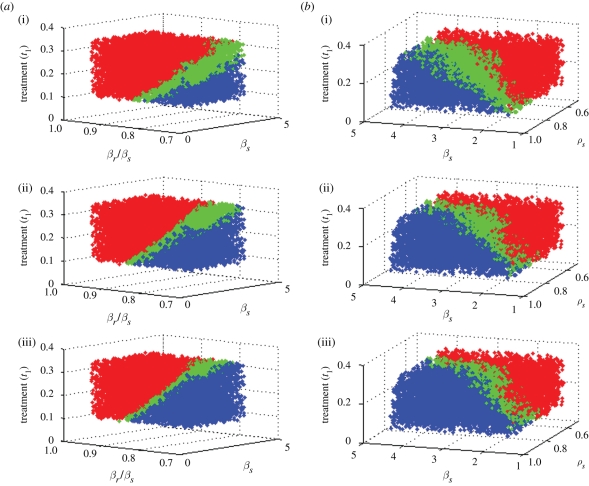
Scatter plots of simulation outcomes at equilibrium in the dual transmission model (model E) with two mechanisms for implementing a fitness cost for the resistant strain: (*a*) relative transmissibility *β*_*r*_/*β*_*s*_, and (*b*) probability of transmission of an *S* infection upon dual transmission, *ρ*_*s*_ > 1/2. Results are shown for three values of *ρ*_single_, the probability that a single infection will be transmitted from a dually infected host, rather than a dual infection, given that a transmission will occur: (i) *ρ*_single_ = 0.1; (ii) *ρ*_single_ = 0.5; (iii) *ρ*_single_ = 0.9. Green, coexistence; red, all disease is strain *R*; blue, all disease is strain *S*.

For model E, which includes the possibility of transmitting both strains simultaneously, there are two distinct approaches to model a fitness cost of drug resistance, and these are shown in figure 3. One is to model *β*_*r*_ < *β*_*s*_ (resistance associated with lower transmissibility) as we have done for the other models. The other is to model *ρ*_*s*_ > 1/2, which implies that when a dually infected individual transmits a single infection, it is more likely that the sensitive strain will be transmitted than the resistant one. We examine the effect of each mechanism, and also vary the probability of single versus dual transmission from those in the dually infectious class, choosing *ρ*_single_ = 0.1, 0.5 and 0.9. Results are illustrated in figure 3, and the numbers of simulations exhibiting coexistence are given in table 2. We find that two factors increase the level of coexistence. One is to increase the chance of simultaneous dual transmission, which is done through lowering the value of *ρ*_single_. The other is to implement the fitness cost of resistance by increasing the chance of transmission of strain *S* in the case that a dually infected individual transmits a single infection (i.e. *ρ*_*s*_ > 1/2). When both of these choices are made, there is a notable coexistence-promoting effect: almost 30 per cent of the simulations show long-term stable coexistence of strains (table 2).

**Table 2.** Probability of long-term coexistence in the simultaneous dual transmission model.

	fitness through *β*_*r*_ < *β*_*s*_ (%)	fitness through *ρ*_*s*_ >1/2 (%)
*ρ*_single_ = 0.1	21	29
*ρ*_single_ = 0.5	20	21
*ρ*_single_ = 0.9	17	16

### 4.2. Impact of strain-specific immunity

Classical Lotka–Volterra models indicate that when there is more intraspecific competition (each species inhibiting its own growth) than interspecific competition (each inhibiting the other's growth), stable coexistence can result (Begon *et al*. 1990). This suggests that changing the ability of each strain of infection to repress its own reproductive success, when compared with its inhibition of the spread of the other strain, will be a coexistence-promoting mechanism that is distinct from the mechanisms we have assessed so far. If it is biologically plausible, one may wish to examine this as a mechanism that promotes coexistence between two biologically distinct strains. There are two major ways in which a particular strain, when present, would tend to compete more strongly with that same strain than with other strains. The first is specialization of strains, for example, if each strain had an affinity for a different host receptor or anatomic site; in this case, colonization of an already-colonized host by a different strain would tend to be easier than colonization by another population of the same strain that is already present. Second, if each strain elicits—while present in the nasopharynx—an immune response that tends to inhibit itself more than the other strain, then once more a host would be more open to acquisition of a strain that is not present than to recolonization by another copy of a strain that is already present. Both of these mechanisms are in effect forms of within-host, strain-specific niche differentiation, and we will use the shorter term ‘niche differentiation’ to describe such mechanisms in general. In this section, we consider the effects of niche differentiation on coexistence.

In our simple model, an individual infected with strain *S* can be reinfected with strain *R*, leading to dual colonization; the presence of a strain already colonizing a host reduces the rate at which this happens, by a proportion (1 − *k*), relative to the rate of primary infections in non-colonized persons. Thus, the transition term from *I*_*S*_ to *I*_*R*_ is *kβ*_*r*_(*I*_*R*_ + *qI*_*SR*_)*I*_*S*_. In that model, we also assume that a host colonized with both strains may be colonized again by one of the strains, ‘knocking out’ the other and sending the host back into the singly colonized. It is assumed there that such knocking-out occurs at the same rate as secondary infection, with half of these events being ‘invisible’ because the incoming and outgoing strains are the same (*c* = 1/2). Thus, the transition term from *I*_*SR*_ to *I*_*S*_, for example, is *ckβ*_*s*_(*I*_*S*_ + *qI*_*SR*_)*I*_*SR*_. However, if it were the case that individuals already colonized with, for example, strain *S* are less likely to acquire another strain *S* than to acquire strain *R*, and vice versa (owing to niche differentiation), then these terms for knocking-out in hosts colonized with both *S* and *R* would be reduced, e.g. *ck*_o_*β*_*s*_(*I*_*S*_+*qI*_*SR*_)*I*_*SR*_, with *k*_o_ < *k*.

We repeat the simulations presented above, but with *k*_o_ = *fk*, for *f* =(1/2, 5/8, 3/4, 7/8); to facilitate comparison between probabilities of coexistence over the four new values of *f*, we used parameter sets that were identical (for all parameters common to the models) with the exception of this single variable. Table 3 summarizes the results, including those for *f* = 1. We do not consider model D here because a distinctive feature of model D is that it does not permit a strain to reinfect a host already carrying that strain, so self-immunity is not an issue.

**Table 3.** Per cent of simulations coexisting in the models when self-protection is greater than cross-protection.

*f*	1/2	5/8	3/4	7/8	1
model A: simple	69	59	47	32	16
model B: treated class	62	53	42	31	19
model C: day care	80	73	62	43	20
model E: dual transmission	79	71	61	47	29

Increasing strain-specific immunity is associated with a marked increase in the possibility of stable coexistence in all the models.

While the day-care model and the dual transmission model reach similar, and high, levels of coexistence when *k*_o_ = *k*/2, the dual transmission model does so while remaining relatively neutral in terms of how much resistance there is overall in the simulations, while the day-care model substantially promotes resistance in this case. Figure 5 in appendix A presents this difference visually through histograms of the fraction of infection that is resistant in the simulations, analogous to those presented in figure 3.

**Figure 4.**
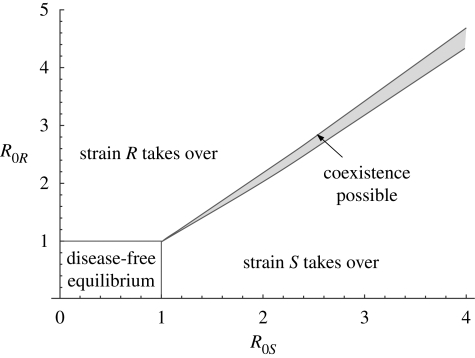
Bifurcation diagram for the simple model, showing the regions of stability of the four equilibria: no disease, strain *S* or *R* takeover and stable coexistence.

**Figure 5.**
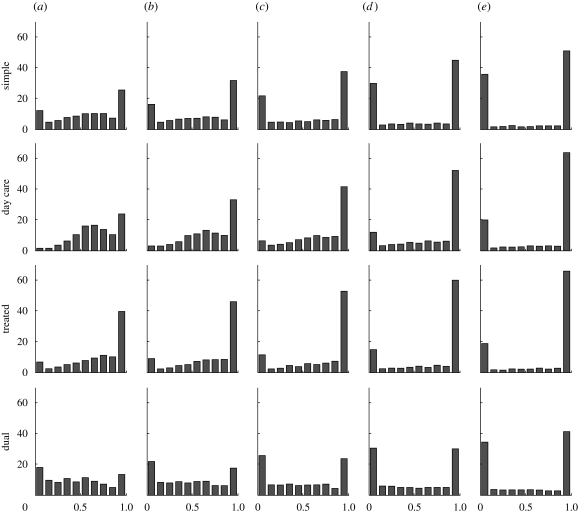
Histograms of the per cent of simulations with coexistence at equilibrium in each model (with the exception of model D), with varying values of self-immunity compared with cross-immunity. (*a*) *k*_*S*_/*k* = 0.5; (*b*) *k*_*S*_/*k* = 0.625; (*c*) *k*_*S*_/*k* = 0.75; (*d*) *k*_*S*_/*k* = 0.875; (*e*) *k*_*S*_/*k* = 1.

## 5. Discussion

The mechanisms that maintain intermediate levels of drug resistance among pneumococcal isolates in diverse geographical areas—which differ substantially in both the prevalence of infection and the usage of antibiotics—are not well understood. The simplest dynamic models that consider strict competition between strain types (in the absence of acquired or imported resistance) predict either the complete takeover or the disappearance of resistance; the strain that eventually dominates is determined by the balance of fitness costs imposed by resistance and the selective pressure of antibiotics. The failure of these simple models to account for the long-term coexistence of drug-resistant and drug-sensitive isolates of *S. pneumoniae* indicates that more realistic models are necessary to explain how phenotypic variants are maintained within a single population of hosts.

One such model explicitly includes a class of individuals on antibiotic therapy; hence there was a protected niche in which drug-resistant strains could flourish even if they were outcompeted among the larger population of hosts who were not currently on treatment (Austin & Anderson 1999). On the ecological principle that habitat heterogeneity leads to coexistence of competitors via specialization (Tilman 1982), we hypothesized that insular subpopulations of individuals, such as children in day care who are in close contact and also highly exposed to commonly used antibiotics, offer a refuge for relatively ‘unfit’ resistant strains that would be outcompeted if mixing between all individuals was random and antibiotic pressure was equally applied. Finally, we suspected that if there were two levels of competition, within a host and in the transmitting host population, specialization might occur and permit coexistence, as has been seen in infectious disease models in other contexts (Levin & Pimentel 1981).

In previous work (Lipsitch *et al*. 2009), we argue that models for exploring mechanisms of coexistence should not artificially facilitate strain coexistence by incorporating implicit mechanisms that protect strains from elimination. Instead, we advocate the use of structurally neutral models, which do not permit asymptotically stable coexistence of indistinguishable strains, as a basis on which mechanisms that may promote coexistence (such as host population heterogeneity or within- versus between-host tradeoffs) can be layered. Here, we follow this advice, starting from models that are neutral when applied to indistinguishable strains and then asking whether they typically permit stable coexistence of sensitive and resistant strains.

We have used the portion of simulations exhibiting long-term coexistence as a metric for how robust coexistence is in a given model structure. Of course, this portion will depend on the distribution from which parameter values are drawn. We have modelled parameters being independent and uniformly distributed over ranges informed where possible by empirical observations; the parameters were taken from the same ranges in each model and models were constructed as much as possible so that a parameter has the same meaning in each model. Thus, we would argue that the comparative conclusions of our work regarding which model structures have the most robust coexistence are to some extent independent of the parameter distributions so long as these are held fixed.

One could take an alternative approach; for example, consider model A and figure 4 in appendix A. Here, coexistence would only be a generic outcome if parameters were taken from a distribution supported primarily on the thin band in parameter space where there is stable coexistence. However, in light of factors such as variation in treatment programmes and antibiotic usage in different settings, contact and host heterogeneity and other factors, it seems less likely that parameter values would be so tightly constrained and interdependent than that there is a model structure for which coexistence is a more robust outcome. It is this comparison between model structures that we hope to elucidate with our approach.

We find, first, that stable coexistence is possible in a simple model of single and dual colonization (model A), even when it is designed to meet the criteria for a structurally neutral model. However, the proportion of parameter sets for which stable coexistence is found is only 16 per cent, which suggests that coexistence is not a typical outcome for such a model. In the treated class model (model B), we had hypothesized that a treated class would provide a reservoir of susceptible individuals accessible only to the resistant strain, thus preventing competitive exclusion of this less fit strain and increasing the possibility of coexistence. While the proportion of parameter sets permitting coexistence was slightly higher (19%), the major effect of including a treated class was to promote resistance, by moving susceptible individuals to states where they are only available to the resistant strain, thus affecting the competition for hosts between the two strains. This effect is clearly visible from a comparison of the histograms in figure 2*e*,*f*, which shows many more parameter sets generating a ‘resistant-only’ outcome in model B.

The day-care model, model C, includes two subpopulations that differ in two ways—in their rates of contact and in their rates of treatment. We hypothesized that such heterogeneity would lead to a greater likelihood of coexistence, with resistant and sensitive strains specializing in the high- and low-treatment populations, respectively. As an extreme case, one can imagine that the population is split into two groups: one in which one strain takes over and a second in which the other strain takes over. This could occur, for example, if the basic reproductive number of the sensitive strain is larger than that of the resistant strain in the general population, but in the day-care population, owing to treatment, the basic reproductive number of the resistant strain is higher. If there were no mixing between groups, this situation would result in coexistence of strain types if each strain can exclude the other in only one of the two non-intermixing subpopulations. Indeed, in model C, coexistence is promoted to some degree when mixing is strongly assortative, but even a small amount of mixing between the groups tends to counteract the effects of host heterogeneity. In summary, model C slightly promotes coexistence, but only when mixing between groups is very low. Compared with model A, model C predicts more success for the resistant strain (figure 2*c*,*g*), just as with model B. In contrast, model D, which allows superinfection and subsequent within-host takeover only by the more fit drug-sensitive strain, has little coexistence-promoting effect, but does favour the sensitive strain relative to model A (figure 2*d*,*h*).

The most robust coexistence among our initial models is found in model E, which allows for simultaneous dual transmission of infections, so that a susceptible individual may obtain both an *S* and an *R* infection directly upon contacting a dually infected individual. In this model, it is possible to obtain comparatively high frequencies of stable coexistence. While this depends on how the fitness costs associated with resistant phenotypes are modelled, and also relies on a reasonably high likelihood of simultaneous dual transmission, it occurs naturally in a model that meets the criteria for biologically identical strains without introducing coexistence of indistinguishable strains for free.

Most favourable to stable coexistence is the assumption that drug-sensitive and drug-resistant strains compete more strongly with themselves than with one another. The choice to have self- and cross-immunity differ depends on the two strains being substantially different in their immunogenic properties, and hence does not fit the framework of structurally neutral models, despite the fact that it can be applied symmetrically to the strains. The question, then, is whether such a mechanism is biologically plausible. We are aware of no evidence that drug-resistance determinants themselves provide protective immunity to pneumococcal colonization or otherwise define niche differentiation. However, drug resistance does tend to be associated with particular serotypes of *S. pneumoniae* (McGee *et al*. 2001; McCormick *et al*. 2003). Serotype-specific immunity to pneumococcal carriage has been documented for some serotypes (Goldblatt *et al*. 2005; Hill *et al*. 2008; Weinberger *et al*. 2008), and there is considerable evidence for the biological and epidemiological heterogeneity of serotypes (Hausdorff *et al*. 2005), which may in part lead to (or reflect) niche differentiation. Thus, it is biologically possible that resistant strains and sensitive strains will, on average, compete more strongly with themselves than with one another.

In this study, we have considered and rejected several mathematical models solely based on the qualitative criterion of coexistence between sensitive and resistant strains of bacteria. Only two of the models considered showed coexistence in at least 20 per cent of sampled parameters, a rather generous criterion for calling coexistence a ‘typical’ outcome; this fraction was much higher if strain-specific immunity was considered. Further work should allow us to further constrain the range of plausible models—thereby improving our biological understanding—by considering additional quantitative aspects of pneumococcal surveillance data. Overall resistance levels correlate well with country-wide antibiotic usage statistics (Goossens *et al*. 2005), and vary seasonally in phase with antibiotic usage, which peaks in winter months (Dagan *et al*. 2008). Future work should aim to replicate quantitatively this high degree of spatial and temporal correlation between antibiotic consumption and the frequency of resistance.

Other factors, including spatial heterogeneity, tradeoffs in transmissibility and virulence, contact structure, heterogeneous host susceptibility and mechanisms for sustained non-equilibrium coexistence, are all likely to play a role in maintaining strain diversity. Furthermore, different pathogens will probably be differently affected by such mechanisms; there is no unique mechanism for the promotion of coexistence. However, the results presented here indicate that the within-host interactions between the strains, in particular the dynamics of coinfection, multiple infections and strain replacement, have a marked effect on the population-level strain composition and the possibility of long-term coexistence.

## Appendix A

Bifurcation diagram for the simple model and histograms of the per cent of simulation with coexistence at equilibrium in each model are shown in figures 4 and 5.
